# Food triggers and inherited metabolic disorders: a challenge to the pediatrician

**DOI:** 10.1186/s13052-018-0456-2

**Published:** 2018-01-25

**Authors:** Evelina Maines, Annunziata Di Palma, Alberto Burlina

**Affiliations:** 1 FDepartment of Women’s and Children’s Healthses, Department of Women’s and Children’s Health, Azienda Provinciale per i Servizi Sanitari, 38122 Trento, Italy; 2 Division of Inherited Metabolic Diseases, Reference Centre Expanded Newborn Screening, Department of Women’s and Children’s Health, University Hospital, Padova, Italy

**Keywords:** Inherited metabolic disorders, Late-onset presentation, Food triggers, Adverse food reactions

## Abstract

Several disorders should be considered in the case of newborns and infants experiencing acute or recurrent symptoms after food ingestion. Immune-mediated adverse food reactions are the most frequent and always to be considered. Nevertheless, in the extensive differential diagnosis, clinicians should also include inherited metabolic disorders (IMDs).

This review reports clinical features and diagnostic aspects of the most common IMDs that may present with acute manifestations triggered by food intake. Major focus will be amino acid and protein metabolism defects and carbohydrate disorders.

Nowadays, for many of these disorders the risk of an acute presentation triggered by food has been decreased by the introduction of expanded newborn screening (NBS). Nevertheless, clinical suspicion remains essential because some IMDs do not have still reliable markers for NBS and a false negative screening result may occur.

The aim of this review is to help pediatricians to take these rare inherited disorders into account in the differential diagnosis of acute or recurrent gastrointestinal symptoms related to food intake, which may avoid delayed diagnosis and potentially life-threatening consequences.

## Background

Several disorders should be considered in the case of newborns and infants experiencing acute or recurrent symptoms after food ingestion: motility disorders or anatomic abnormalities of the gastrointestinal tract, infections, systemic diseases and adverse food reactions are the most frequent and always to be considered [1].

**Fig. 1:**
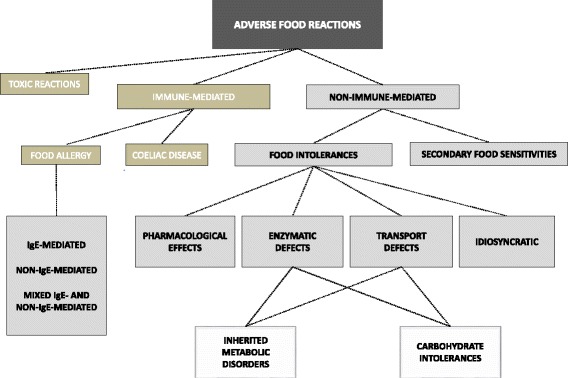
Classification of adverse reactions to food [Adapted from: Turnbull et al. [3]]

Adverse reactions to food, excluding toxic reactions, are distinguished on the basis of the pathogenetic mechanism and may be either immune-mediated or non-immune-mediated (Fig. 1). Immunological food reactions are the most common, including IgE-mediated and non-IgE-mediated food allergies, and coeliac disease. Nevertheless, non-immunological food reactions, such as secondary food sensitivities and food intolerances, may also play an important role [2–4].

Adverse food reactions due to secondary food sensitivities occur with or after the effects of other conditions (e.g., secondary to gastrointestinal disorders or secondary to drug treatment) [4].

Food intolerances can originate from the pharmacological effects of vasoactive substances present in foods (e.g., histamine) or from enzymatic or transport defects. In rare cases, the pathogenetic mechanism remains undefined, and the reactions are classified as idiosyncratic [3, 5].

Enzymatic or transport food intolerances can occur due to defects of enzymes or transporters specifically located in the digestive system (e.g., carbohydrate intolerances) [5] or due to systemic defects of enzymes or transporters of specific metabolic pathways (inborn errors of metabolism) [6].

Inherited metabolic disorders (IMDs) are a complex and heterogeneous group of rare monogenic disorders, usually resulting from a deficient activity in a single pathway of intermediary metabolism [7].

Each IMD is individually rare, but data from expanded newborn screening (NBS) programs outlines an estimated incidence of 1:1500–5000 [8, 9].

Although the most severe forms present usually in the neonatal period, late-onset presentations may represent an important cause of morbidity and mortality in the pediatric age [10].

Food may be a specific trigger of metabolic decompensation at any age.

This review reports clinical features and diagnostic aspects  of the most common IMDs that may present with acute manifestations triggered by food intake. Major focus will be amino acid and protein metabolism defects and carbohydrate disorders.

The aim of this review is to help pediatricians to take these rare inherited disorders into account in the differential diagnosis of acute or recurrent gastrointestinal symptoms related to food intake, which may avoid delayed diagnosis and potentially life-threatening consequences.

### Disorders triggered by proteins

#### Case presentation 1

*The patient was born at term after an uneventful pregnancy from two unrelated parents. The baby was exclusively breast-fed until six months of age. Soon after the start of weaning, between the ages of 6 and 12 months, he experienced repetitive vomiting and episodes of diarrhea, approximately within 1 to 3 h after the consumption of meat, chicken, fish, and egg. Since symptoms always recurred after the administration of the same foods, an allergic problem was suspected. Food protein-induced enterocolitis (FPIES), a non-IgE-mediated gastrointestinal disorder, was diagnosed. Protein-rich foods related to the symptoms were eliminated from his diet and were not subsequently reintroduced because the patient manifested a severe aversion to these foods.*

*At 7 years, clinical examination revealed short stature, mild language retardation and hepatosplenomegaly. Laboratory tests showed high serum ferritin (770 ng/ml), and lactic dehydrogenase (LDH) values (1007 UI/L) with normal white blood cell, erythrocyte and platelet counts.*

*Other laboratory tests (including blood gases and ammonia) were all in the normal range.*

*Bone marrow aspirate examination revealed evidence of erythrophagocytosis (HP).*

*Then, due to the clinical features and the dietary history of recurrent vomiting and spontaneous protein-rich food aversion, plasma and urinary amino-acid analysis were performed.*

*We found lower than normal plasmatic levels of lysine, ornithine, and arginine, and higher than normal urinary levels of the same amino acids. Based on the results, we diagnosed lysinuric protein intolerance (LPI). The diagnosis was confirmed by molecular analysis of SLC7A7 gene. The patient started a controlled low-protein diet and an oral treatment with sodium benzoate and citrulline* [11].

#### Case presentation 2

*A male 15 months of age was admitted to the hospital after a day of persistent vomiting and somnolence alternating with periods of irritability. He had a history of recurrent vomiting by the 6 months of age. According to his parents, the child had no spontaneous aversion to proteins (daily intake 2.5 g/kg) and the episodes seemed to be more related to fructose ingestion even the child used to have a meal including fruit and meat together. Routine biochemical investigations performed in well-state showed elevated transaminases (AST 300 U/L, ALT 1727 U/L), normal ammonia, lactate, amino acids and acylcarnitine profiles. Isoelectric focusing (IEF) of serum transferrin (Tf) showed an increased trisialo-Tf form, suggesting a possible diagnosis of fructosemia.*

*The patient was put on a low-fructose diet and molecular analysis for aldolase B gene mutation was performed.*

*About 4 months later, he was readmitted to the hospital with severe acute encephalopathy after a day of continuous vomiting. Vomiting was started by the ingestion of a sandwich with ham.*

*Brain CT scan showed severe edema. Liver transaminases were increased (AST 557 U/L, ALT 1923 U/L); ammonia reached a value of 600 μmol/L. Despite intensive treatments, the child died the following morning. Plasma amino acids and urinary organic acids performed during acute symptoms were suggestive for ornithine transcarbamylase deficiency (OTCD). OTC gene analysis confirmed the diagnosis* [12]*.*

Deficiencies of enzymes or transporters involved in amino acid and/or protein metabolism may present acutely in the neonatal period, typically after a short symptom-free interval, or later in life with acute, intermittent or progressive forms. In all cases, clinical features become apparent as a result of the accumulation of toxic compounds proximal or related to the metabolic block (*intoxication type disorders*) [10]. The most common acute (or recurrent) signs of “intoxication” are vomiting or feeding difficulties with lethargy, encephalopathy that may rapidly progress to coma, and liver failure [10].

Triggers provoking acute metabolic attacks are more commonly catabolic states (e.g., prolonged fasting, infections, fever, surgery, chemotherapy, high-dose glucocorticoids). Nevertheless, an increased protein intake (e.g., weaning dietary changes, a barbecue) may also be a cause of metabolic decompensation both in children and in adults [13].

Adult-onset cases unmasked by parenteral nutrition have also been described [14, 15].

Acute manifestations triggered by protein-rich food intake may be observed in patients with urea cycle disorders (UCDs) [13, 16, 17], lysinuric protein intolerance (LPI) [11, 18], organic acidemias (OAs) [16, 19], and maple syrup urine disease (MSUD) [20] (Table 1). Intoxication is caused by ammonia (UCDs, LPI), toxic organic acids (OAs) or amino acids and related toxic compounds (MSUD). Hyperinsulinism/hyperammonemia syndrome (HI/HA) may be included in the group of disorders triggered by proteins, because hypoglycemias are usually unmasked by protein-rich meals (leucine sensitivity) [21–23]. Nevertheless, HI/HA does not belong to intoxication type disorders group, being a very rare form of congenital hyperinsulinism (CHI) [24].

**Table 1:** Diagnostic features and management of the most common IMDs triggered by proteins

	Disorders triggered by proteins				
	UCDs	LPI	OAs	MSUD	HI/HA
*Newborn screening*	Distal defects	No	Yes	Yes	No
*Food triggers*	Protein load	Protein load	Protein load	Protein load	Protein load (leucine sensitivity)
*Age of onset*	Variable. From a few days after birth (complete enzymatic deficiencies) to adult age (partial enzymatic deficiencies)	Variable. Common during weaning	Variable. From a few days after birth (complete enzymatic deficiencies) to adult age (partial enzymatic deficiencies)	Variable. From a few days after birth (complete enzymatic deficiencies) to adult age (partial enzymatic deficiencies)	After the first few months of life
*Main presenting features*	Acute or episodic encephalopathy with lethargy and vomiting, liver failure, spontaneous protein aversion	Recurrent emesis and/or diarrhea, episodes of postprandially altered mental status. Strong aversion to high-protein foods by the age of 1 year	Acute or episodic encephalopathy with lethargy and vomiting	Acute or episodic encephalopathy with lethargy and vomiting. Maple syrup odor	Recurrent episodes of profound hypoglycemia induced by fasting and protein-rich meals
*Main routine laboratory findings*	Hyperammonemia	Postprandial hyperammonemia, high levels of LDH and ferritin, hypertriglyceridemia	Ketoacidosis, hyperammonemia, hyperlactatemia	Ketoacidosis	Persistent mild or moderate hyperammonemia, recurrent hypoketotic hypoglycemia
*Diagnostic confirmation*	Plasma amino acid analysis, urinary orotic acid dosage. Genetic testing	Plasma and urinary amino acid analysis. Genetic testing	Urinary organic acid analysis, plasma acylcarnitine profile. Genetic testing	Plasma amino acid analysis, urinary organic acid profile. Genetic testing	Genetic testing
*Acute management*	Specialist centre. Stop protein intake, ammonia detoxification, measures to reverse catabolism	Specialist centre. Stop protein intake, ammonia detoxification, measures to reverse catabolism	Specialist centre. Stop protein intake, ammonia detoxification, measures to reverse catabolism	Specialist centre. Stop protein intake, leucine and BCKAs detoxification, measures to reverse catabolism	Specialist centre. Prompt correction of hypoglycemia
*Chronic management*	- Protein-restricted diet - adequate energy intake - ammonia scavengers - oral arginine or citrulline supplementation - liver transplantation for selected patients	- Protein-restricted diet - adequate energy intake - ammonia scavengers - oral lysine and citrulline supplementation	- Protein-restricted diet - adequate energy intake - ammonia scavengers - defect-specific amino acids supplementation - liver/kidney transplantation for selected patients	- Protein-restricted diet - adequate energy intake - oral isoleucine and valine supplementation - liver transplantation for selected patients	- Protein-restricted diet - oral diazoxide
*Natural history*	Variable. The duration and severity of hyperammonemia strongly correlate with brain damage	Variable. The duration and severity of hyperammonemia strongly correlate with brain damage. Late complications may be fatal	Variable. The duration and severity of acidosis and hyperammonemia strongly correlate with brain damage. Late complications may be fatal	Variable. The duration and severity of coma strongly correlate with brain damage	Variable. The duration and severity of hypoglycemias strongly correlate with brain damage. Increased frequency of generalized seizures

**Fig. 2:**
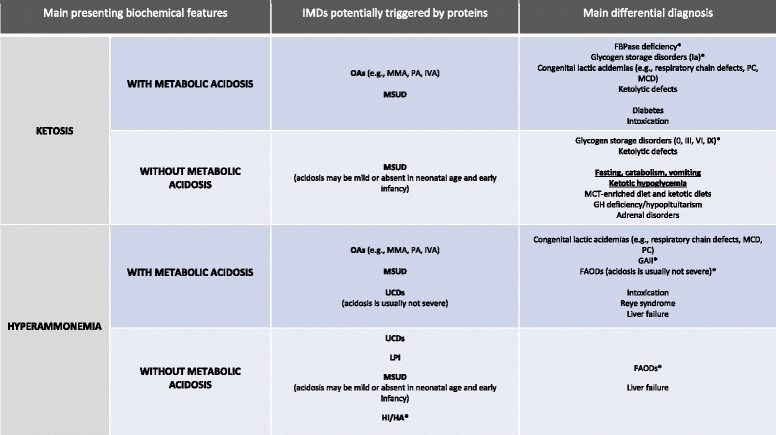
Diagnostic algorithm to guide the initial approach to IMDs triggered by proteins, based on the main presenting biochemical features (ketosis and hyperammonemia with or without metabolic acidosis). IMDs not triggered by proteins but with similar biochemical features are shown as differential diagnosis. Abbreviations: *FAODs* fatty acid oxidation defects, *FBPase deficiency* fructose-1,6-bisphospatase deficiency, *GAII* glutaric acidemia type II, *GH* growth hormone, *HI/HA* hyperinsulinism hyperammonemia syndrome, *IVA* isovaleric acidemia, LPI lysinuric protein intolerance, *MCD* multiple carboxylase deficiency, *MCT* medium-chain triglycerides, *MMA* methylmalonic acidemia, *MSUD* maple syrup urine disease, *OAs* organic acidurias, *PA* propionic acidemia, *PC* pyruvate carboxylase, *UCDs* urea cycle disorders. * hypoglycemia is usually the main presenting symptom

Based on few laboratory data (ketones, ammonia, blood gas analysis) collected at the same time during the acute attack, we propose a schematic diagnostic algorithm to guide the initial approach and the differential diagnosis of acute presentations of IMDs triggered by proteins (Fig. 2) (Table 1).

Nowadays, for many of these IMDs the risk of an acute presentation triggered by food has been decreased by the introduction of expanded NBS.

NBS may detect distal UCDs *(citrullinemia type 1 or argininosuccinate synthetase deficiency [ASSD, OMIM #215700], argininosuccinic aciduria or argininosuccinate lyase deficiency [ASLD, OMIM #207900] and arginase 1 deficiency or argininemia [ARG1D, OMIM #207800])*, and citrullinemia type 2 or citrin deficiency (Citrin-D, OMIM #605814 and #603471), a mitochondrial transport defect of the urea cycle.

Proximal enzymatic defects of the urea cycle *(carbamoylphosphate synthase 1 deficiency [CPS1D, OMIM #237300], ornithine transcarbamylase deficiency [OTCD, OMIM #311250] and N-acetylglutamate synthase deficiency [NAGSD, OMIM #237310])*, and lysinuric protein intolerance (LPI, OMIM #222700) are not usually included in the screening panel, because of the low specificity and sensitivity of NBS marker (hypocitrullinemia) [25]. However, screening metabolites kits including orotic acid and/or glutamine assay have been proposed to identify patients with OTCD, as well as with other UCDs [26, 27].

NBS may also detect OAs *(*e.g.*, propionic acidemia [PA, OMIM #606054], methylmalonic acidemia [MMA, OMIM #251000], isovaleric acidemia [IVA, OMIM #243500] and glutaric acidemia type 1 [GA1, OMIM #**231670**]* and maple syrup urine disease [MSUD, OMIM #248600]). OAs result from a defect in the branched-chain amino acids (BCAAs) or lysine catabolism [28–30], leading to a specific NBS acylcarnitines pattern [31–33]. MSUD is also due to a defect in the metabolic pathway of the BCAAs [20], which leads to high levels of BCAAs and alloisoleucine [34].

### Disorders triggered by sugars

#### Case presentation

*A female infant was exclusively breastfed until 6 months of age. At 7 months she underwent a normal weaning, but she developed recurrent vomiting. The parents gradually eliminated from her diet several foods, in particular mousse fruits, because the patient seemed to reject them.*

*At 12 months, the patient had resumed a predominantly milk diet and presented a growth below the 3rd percentile. Her pediatrician suggested biochemical exams and abdominal ultrasound. Blood analyses showed a mild increase of liver enzymes (ALT 98 U/L AST 75 U/L), with normal liver function and no cholestasis.*

*No sign of malabsorption or gastrointestinal disease was found.*

*The ultrasonography of the abdomen showed hepatomegaly (the lower margin was 5 cm below the lower pole of the right kidney) and hepatic steatosis.*

*The patient was brought to the pediatric department of a tertiary care institution for evaluation. Blood exams revealed no significant abnormalities with the exception of hypertransaminasemia (ALT 114 U/L, AST 113 U/L). The histological analysis of liver biopsy revealed macrovesicular steatosis, mild fibrosis, and no evidence of inflammation, necrosis or bile stasis.*

*IEF of serum Tf was normal.*

*Due to the dietary history suggestive for fructosemia, ALDOB gene analysis was performed. The exam revealed the homozygous mutation p.N335 K and confirmed the diagnosis.*

*A fructose-, sorbitol- and sucrose-free diet was immediately started and there was a complete recovery of the blood abnormalities and a significant reduction of liver size (not published).*

Intoxication type disorders triggered by sugars include galactosemia and fructosemia, also known as hereditary fructose intolerance (HFI, OMIM #229600) (Table 2). Intoxication is caused by toxic carbohydrate metabolites derived by exogenous intake of galactose and fructose, respectively [35, 36].

**Table 2:** Diagnostic features and management of the most common IMDs triggered by sugars

	Disorders triggered by sugars		
	CG	Generalized GALE	HFI
*Newborn screening*	Yes	Yes	No
*Food triggers*	Breast milk, infant formulas and foods containing galactose or lactose	Breast milk, infant formulas and foods containing galactose or lactose	Fructose-, sucrose-, and sorbitol- containing foods
*Age of onset*	Within a few days after breastfeeding or when lactose-containing formula feeding is started	Within a few days after breastfeeding or when lactose-containing formula feeding is started	At the time of weaning or after supplementary food
*Main presenting features*	Poor feeding, vomiting, hepatomegaly, jaundice, liver failure, sepsis, cataracts	Poor feeding, vomiting, hypotonia, hepatomegaly, jaundice, liver failure, cataracts	Vomiting, postprandial hypoglycemia, progressive liver dysfunction, aversion to fructose-containing foods and sweets
*Main routine laboratory findings*	Liver damage, increased plasma galactose, urinary reducing substances	Liver damage, increased plasma galactose, urinary reducing substances	Hypoglycemia, urinary reducing substances. Metabolic acidosis, liver and kidney damage in severe cases
*Diagnostic confirmation*	Erythrocyte GALT enzyme activity, erythrocyte galactose-1-phosphate concentration. Genetic testing	Erythrocyte GALE enzyme activity, erythrocyte galactose-1-phosphate concentration. Genetic testing	IEF of Tf. Genetic testing
*Acute management*	Specialist centre. Lactose-free infant formula	Specialist centre. Lactose-free infant formula	Specialist centre. Prompt correction of hypoglycemia
*Chronic management*	Lactose-free, galactose-restricted diet throughout life	Lactose-free, galactose-restricted diet throughout life.	Fructose-, sucrose-, and sorbitol-restricted diet. Vitamin C supplementation
*Natural history*	Extreme variability in long-term outcome. Dyspraxias, learning disabilities, mental retardation, ataxia, tremors, and premature ovarian insufficiency in females may be present	Limited long-term outcome data. No evidence of premature ovarian insufficiency in females	Benign disease if appropriately diagnosed and treated

HFI usually presents at the time of weaning, when fruits and vegetables are introduced into the diet. The patients are asymptomatic as long as they avoid foods containing fructose or any of its common precursors such as sucrose (a disaccharide composed of fructose and glucose, also known as table sugar) and sorbitol (present in natural products, especially in dried fruits, and added to others as sweeteners). Acute symptoms include gastrointestinal discomfort, feeding difficulties, vomiting, pallor, metabolic acidosis, hepatomegaly, hypoglycemia, restlessness, lethargy and shock. Prolonged fructose ingestion can ultimately lead to an aversion towards fructose-containing foods, a strong distaste for sweet food, failure to thrive, liver failure and renal dysfunction [37, 38].

In the case of galactosemia, breastfeeding or lactose-containing formula feeding during the first days of life cause severe liver dysfunction, manifested as jaundice, hepatomegaly, hypoglycemia and coagulation disturbances, and gastrointestinal findings of poor feeding, vomiting and diarrhea. The onset of illness may be acute and fulminant and may often be confused with neonatal sepsis due to the *E. coli* infection [39, 40].

The most prominent features of both defects in acute phase are hypoglycemia and hepatomegaly, even though late-onset forms of HFI may rarely present with isolated hypoglycemia [10].

**Fig. 3:**
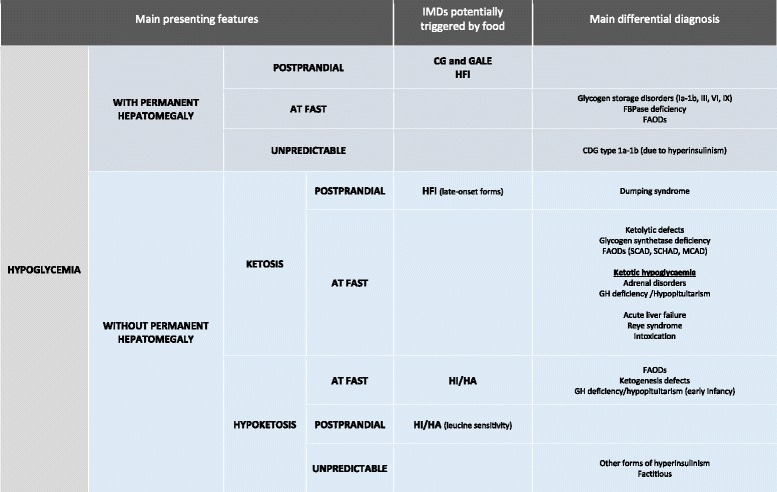
Diagnostic algorithm to guide the initial approach to hypoglycemia, based on the liver size and the timing of hypoglycemia. Postprandial hypoglycemias of HFI and galactosemias differentiate these disorders from others characterized by fasting or unpredictable hypoglycemias. Hypoglycemias in HI/HA are induced both by fasting and by protein-rich meals (leucine sensitivity). Disorders with similar presenting features are shown as differential diagnosis. Abbreviations: *CDG* congenital defect of glycosylation, *FAODs* fatty acid oxidation defects, *FBPase deficiency* fructose-1,6-bisphospatase deficiency, *HI/HA* hyperinsulinism hyperammonemia syndrome, *MCAD* medium-chain acyl-CoA dehydrogenase deficiency, *SCAD* short-chain acyl-CoA dehydrogenase deficiency, *SCHAD* short-chain 3-hydroxyacyl-CoA dehydrogenase

Postprandial hypoglycemias of HFI and galactosemia differentiate these disorders from others characterized by fasting or unpredictable hypoglycemias (e.g., fatty acid oxidation defects, glycogenosis, hyperinsulinism) (Fig. 3).

HFI is not included in NBS panel, because of technical difficulties in screening for a condition in which there is no neonatal exposure to the offending agent.

On the contrary, most infants with galactosemia are now diagnosed through routine NBS, even if many patients may present symptoms before referral for abnormal NBS. Screening test is done primarily to detect clinically devastating classic galactosemia (CG, OMIM #230400) due to defective function of galactose-1-phosphate-uridyl transferase (GALT) and generalized forms of epimerase deficiency galactosemia (GALE deficiency galactosemia, OMIM #230350), due to defective function of UDP-galactose-4-epimerase (GALE) [39–41].

## Conclusions

Despite the introduction of NBS for many IMDs potentially triggered by food intake, pediatricians should systematically consider these disorders in the differential diagnosis of acute or recurrent gastrointestinal symptoms related to food ingestion, in particular if they occur as a part of a systemic disorder.

**Fig. 4:**
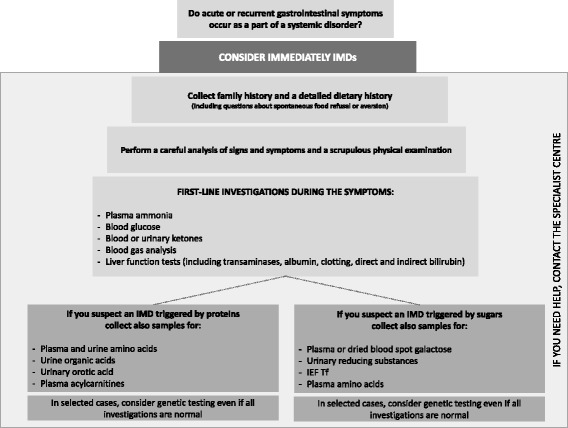
Diagnostic algorithm to suspect and investigate IMDs when acute or recurrent gastrointestinal symptoms occur as a part of a systemic disorder

Once clinical suspicion is aroused, general supportive measures and metabolic laboratory investigations must be undertaken immediately (Fig. 4). Plasma and urine samples should be promptly collected during the acute phase of illness because some tests could be normal during asymptomatic periods.

## Abbreviations

BCAAs: Branched-chain amino acids
CG: Classic galactosemia
GALE: UDP-galactose-4′-epimerase
GALT: Galactose-1-phosphate-uridyltransferase
HFI: Hereditary fructose intolerance or fructosemia
HI/HA: Hyperinsulinism/hyperammonemia syndrome
HP: Erythrophagocytosis
IEF: Isoelectric focusing
IMD(s): Inherited metabolic disorder(s)
LDH: Lactic dehydrogenase
LPI: Lysinuric protein intolerance
MSUD: Maple syrup urine disease
NBS: Newborn screening
OA(s): Organic acidemia(s)
OTCD: Ornithine transcarbamylase deficiency
Tf: Serum transferrin
UCD(s): Urea cycle disorder(s)

## References

[1] Sreedharan R, Liacouras CA. Major symptoms and signs of digestive tract disorders. In: Kliegman R, Stanton B, St. Geme JW, Schor NF, Behrman RE, editors. Nelson textbook of pediatrics. 20th ed. Philadelphia: Elsevier; 2016. p. 1758–68.

[2] Ortolani C, Pastorello EA (2006). Food allergies and food intolerances. Best Pract Res Clin Gastroenterol.

[3] Turnbull JL, Adams HN, Gorard DA (2015). Review article: the diagnosis and management of food allergy and food intolerances. Aliment Pharmacol Ther.

[4] Taylor SL, Hefle SL (2001). Food allergies and other food sensitivities. Food Technol.

[5] Berni Canani R, Pezzella V, Amoroso A, Cozzolino T, Di Scala C, Passariello A (2016). Diagnosing and treating intolerance to carbohydrates in children. Nutrients.

[6] Guandalini S, Newland C (2011). Differentiating food allergies from food intolerances. Curr Gastroenterol Rep.

[7] Zschocke J, Hoffmann GF, Zschocke J, Nyhan WL (2010). Disorders of intermediary metabolism. Inherited Metabolic Diseases: a clinical approach.

[8] Seymour C, Thomason M, Chalmers R, Addison G, Bain M (1997). Newborn screening for inborn errors of metabolism: a systematic review. Health Technol Assess.

[9] Pourfarzam M, Zadhoush F (2013). Newborn screening for inherited metabolic disorders; news and views. J Res Med Sci.

[10] Saudubray JM, Saudubray JM, van den Berghe G, Walter JH (2012). Clinical approach to inborn errors of metabolism in paediatrics. Inborn metabolic diseases. Diagnosis and treatment.

[11] Maines E, Comberiati P, Piacentini GL, Boner AL, Peroni DG (2013). Lysinuric protein intolerance can be misdiagnosed as food protein-induced enterocolitis syndrome. Pediatr Allergy Immunol.

[12] Burlina AB, Peduto A, Di Palma A, Bellizzi A, Sperlì D, Morrone A (2006). An unusual clinical and biochemical presentation of ornithine transcarbamylase deficiency in a male patient. J Inherit Metab Dis.

[13] Häberle J, Boddaert N, Burlina A, Chakrapani A, Dixon M, Huemer M (2012). Suggested guidelines for the diagnosis and management of urea cycle disorders. Orphanet J Rare Dis.

[14] Felig DM, Brusilow SW, Boyer JL (1995). Hyperammonemic coma due to parenteral nutrition in a woman with heterozygous ornithine transcarbamylase deficiency. Gastroenterology.

[15] Benque A, Bommelaer G, Rozental G, Cales P, Cathelineau L, Pham Dinh D (1984). Chronic vomiting in a case of citrullinaemia detected after treatment by total parenteral nutrition. Gut.

[16] Kölker S, Garcia-Cazorla A, Valayannopoulos V, Lund AM, Burlina AB, Sykut-Cegielska J (2015). The phenotypic spectrum of organic acidurias and urea cycle disorders. Part 1: the initial presentation. J Inherit Metab Dis.

[17] Smith W, Kishnani PS, Lee B, Singh RH, Rhead WJ, Sniderman King L (2005). Urea cycle disorders: clinical presentation outside the newborn period. Crit Care Clin.

[18] Cimbalistiene L, Lehnert W, Huoponen K, Kucinskas V (2007). First reported case of lysinuric protein intolerance (LPI) in Lithuania, confirmed biochemically and by DNA analysis. J Appl Genet.

[19] Feinstein JA, O'Brien K (2003). Acute metabolic decompensation in an adult patient with isovaleric acidemia. South Med J.

[20] Strauss KA, Puffenberger EG, Morton DH, Pagon RA, Adam MP, Ardinger HH (1993). Maple syrup urine disease. GeneReviews® [Internet].

[21] Hsu BY, Kelly A, Thornton PS, Greenberg CR, Dilling LA, Stanley CA (2001). Protein-sensitive and fasting hypoglycemia in children with the hyperinsulinism/hyperammonemia syndrome. J Pediatr.

[22] De Lonlay P, Benelli C, Fouque F, Ganguly A, Aral B, Dionisi-Vici C (2001). Hyperinsulinism and hyperammonemia syndrome: report of twelve unrelated patients. Pediatr Res.

[23] Tran C, Konstantopoulou V, Mecjia M, Perlman K, Mercimek-Mahmutoglu S, Kronick JB (2015). Hyperinsulinemic hypoglycemia: think of hyperinsulinism/hyperammonemia (HI/HA) syndrome caused by mutations in the GLUD1 gene. J Pediatr Endocrinol Metab.

[24] Faletra F, Athanasakis E, Morgan A, Biarnés X, Fornasier F, Parini R (2013). Congenital hyperinsulinism: clinical and molecular analysis of a large Italian cohort. Gene.

[25] Cavicchi C, Malvagia S, la Marca G, Gasperini S, Donati MA (2009). Hypocitrullinemia in expanded newborn screening by LC-MS/MS is not a reliable marker for ornithine transcarbamylase deficiency. J Pharm Biomed Anal.

[26] Trinh MU, Blake J, Harrison JR, Gerace R, Ranieri E, Fletcher JM (2003). Quantification of glutamine in dried blood spots and plasma by tandem mass spectrometry for the biochemical diagnosis and monitoring of ornithine transcarbamylase deficiency. Clin Chem.

[27] Held PK, Haynes CA, De Jesús VR, Baker MW (2014). Development of an assay to simultaneously measure orotic acid, amino acids, and acylcarnitines in dried blood spots. Clin Chim Acta.

[28] Baumgartner MR, Hȍrster F, Dionisi-Vici C, Haliloglu G, Karall D, Chapman KA, et al. Proposed guidelines for the diagnosis and management of methylmalonic and propionic acidemia. Orphanet J Rare Dis. 2014;9(130) 10.1186/s13023-014-0130-8 PMC4180313 25205257

[29] Boy N, Mühlhausen C, Maier EM, Heringer J, Assmann B, Burgard P (2017). Proposed recommendations for diagnosing and managing individuals with glutaric aciduria type 1: second revision. J Inherited Metab Dis.

[30] Vockley J, Ensenauer R (2006). Isovaleric acidemia: new aspects of genetic and phenotypic heterogeneity. Am J Med Genet C Semin Med Genet.

[31] Monostori P, Klinke G, Richter S, Baráth Á, Fingerhut R, Baumgartner MR, Kölker S, Hoffmann GF, Gramer G, Okun JG (2017). Simultaneous determination of 3-hydroxypropionic acid, methylmalonic acid and methylcitric acid in dried blood spots: Second-tier LC-MS/MS assay for newborn screening of propionic acidemia, methylmalonic acidemias and combined remethylation disorders. PLoS One.

[32] Minkler PE, Stoll MSK, Ingalls ST, Hoppel CL (2017). Selective and accurate C5 acylcarnitine quantitation by UHPLC-MS/MS: Distinguishing true isovaleric acidemia from pivalate derived interference. J Chromatogr B Analyt Technol Biomed Life Sci.

[33] Tsai FC, Lee HJ, Wang AG, Hsieh SC, Lu YH, Lee MC (2017). Experiences during newborn screening for glutaric aciduria type 1: Diagnosis, treatment, genotype, phenotype, and outcomes. J Chin Med Assoc.

[34] Wasim M, Awan FR, Khan HN, Tawab A, Iqbal M, Ayesha H. Aminoacidopathies: Prevalence, Etiology, Screening, and Treatment Options. Biochem Genet. 2017 Nov 1; 10.1007/s10528-017-9825-6. [Epub ahead of print] 10.1007/s10528-017-9825-6 29094226

[35] Prietsch V, Lindner M, Zschocke J, Nyhan WL, Hoffmann GF (2002). Emergency management of inherited metabolic diseases. J Inherit Metab Dis.

[36] Santer R, Klepper J, Smt GP, Blau N (2014). Disorders of carbohydrate metabolism and glucose transport. Physician’s guide to the diagnosis, treatment, and follow-up of inherited metabolic diseases.

[37] Baker P, Ayres L, Gaughan S, Adam MP, Ardinger HH, Pagon RA (1993). Hereditary fructose intolerance. 2015 Dec 17. GeneReviews® [Internet]..

[38] Steinmann B, Santer R, Saudubray JM, van den Berghe G, Walter JH (2012). Disorders of fructose metabolism. Inborn Metabolic Diseases. Diagnosis and Treatment.

[39] Berry GT, Walter JH, Saudubray JM, van den Berghe G, Walter JH (2012). Disorders of galactose metabolism. Inborn Metabolic Diseases. Diagnosis and Treatment.

[40] Berry GT, Pagon RA, Adam MP, Ardinger HH, Wallace SE, Amemiya A, LJH B (1993). Classic Galactosemia and Clinical Variant Galactosemia. GeneReviews® [Internet].

[41] Fridovich-Keil J, Bean L, He M, Adam MP, Ardinger HH, Pagon RA (1993). Epimerase deficiency Galactosemia. 2011 Jan 25 [updated 2016 Jun 16]. GeneReviews® [Internet].

